# Chromosome-level genome assembly of the two-spotted spider mite *Tetranychus urticae*

**DOI:** 10.1038/s41597-024-03640-2

**Published:** 2024-07-18

**Authors:** Li-Jun Cao, Tian-Bo Guan, Jin-Cui Chen, Fangyuan Yang, Jing-Xian Liu, Feng-Liang Jin, Shu-Jun Wei

**Affiliations:** 1 College of Plant Protection, South China Agricultural University, Guangzhou, China; 2 Institute of Plant Protection, Beijing Academy of Agriculture and Forestry Sciences, Beijing, China

**Keywords:** Genome, Sequencing

## Abstract

The two-spotted spider mite, *Tetranychus urticae* Koch (Acari: Tetranychidae), is a notorious pest in agriculture that has developed resistance to almost all chemical types used for its control. Here, we assembled a chromosome-level genome for the TSSM using Illumina, Nanopore, and Hi-C sequencing technologies. The assembled contigs had a total length of 103.94 Mb with an N50 of 3.46 Mb, with 87.7 Mb of 34 contigs anchored to three chromosomes. The chromosome-level genome assembly had a BUSCO completeness of 94.8%. We identified 15,604 protein-coding genes, with 11,435 genes that could be functionally annotated. The high-quality genome provides invaluable resources for the genetic and evolutionary study of TSSM.

## Background & Summary

The two-spotted spider mite (TSSM), *Tetranychus urticae* Koch (Acari: Tetranychidae), is a notorious agricultural pest, with over 1,100 documented host plants^1^. It causes damage to a wide variety of vegetables, fruit trees, and flowers worldwide. Despite numerous control methods developed to control TSSM, it remains one of the major challenges to mitigating the damage of the TSSM in fields^2–5^. The TSSM has a high potential to adapt to environmental changes^6,7^. It has developed resistance to almost all types of pesticide used to its control^8^. A reference genome is essential for understanding the ecology and genetics of adaptation as well as for developing new control methods of TSSM. A TSSM genome was determined using Sanger sequencing, which is one of the early reported pest genomes^7^. The assembly has a size of 89.6 Mb with 640 scaffolds^7^. It has been widely used and significantly enhanced the studies of TSSM, especially in the fields of pesticide resistance, adaptation to host plants, and environmental changes^9–14^. To improve the continuity of the TSSM genome and correct misassembled scaffolds, Wybouw, *et al*.^15^ assembled the Sanger sequences into three pseudochromosomes by using population allele frequency data and *de novo* assemblies of seven strains from Illumina data. The number of chromosomes is consistent with previous cytological work^16,17^.This chromosome-level genome resolves discontinuities of allele frequencies and facilitates the genome-wide scanning of genes and mutations underlying the evolutionary adaptation of TSSM^15,18,19^.

In this study, we assembled a chromosome-level genome for the TSSM using a combination of Nanopore long-read and Illumina short-read sequencing, Hi-C technology, and RNA-sequencing (RNA-seq). We yielded a nuclear genome assembly of 87.7 Mb, with an N50 of 29.6 Mb and BUSCO (Benchmarking Universal Single-Copy Ortholog) completeness of 93.4%. This high-quality genome will provide invaluable resources for the study of the TSSM and its relative issues.

## Methods

### Materials and sequencing

The TSSM strain used for sequencing was collected from Xiaoshan City of Zhejiang province. To decrease the effect of heterozygosity on subsequent analysis, a lab population was reared on French bean *Phaseolus vulgaris* from a small population (about 200 individuals) for continuous generations (about 20 generations) before sequencing, under 25 ± 1 °C, 60 ± 5% relative humidity and L16: D8 photoperiod. Approximately 200 individuals were used for Illumina, 2000 for NanoPore, and 3000 for Hi-C proximity ligation library construction. About 200 larvae and adults were used for transcriptome sequencing for each of the three libraries. Genomic DNA was extracted using the DNeasy tissue kit (Qiagen, Hilden, Germany) for Illumina library construction and the MagAttract HMW DNA kit (Qiagen, Hilden, Germany) for NanoPore library construction. For the Hi-C library, the genome was digested by the restriction enzyme *DpnII*, and fragments were then sheared into ~400 bp. The Hi-C library was sequenced using the DNBSEQ-T7 platform. RNA-seq libraries were prepared using VAHTSTM mRNA-seq V2 Library Prep Kit (Vazyme, Nanjing, China) and sequenced on the Illumina NovaSeq platform. Sequencing data generated from each library are provide in Table 1.

**Table 1:** Summary statistics of generated sequencing data for *Tetranychus urticae* genome assembly and annotation in this study.

Library	Sequencing instrument	Size (bp)	Coverage	Accession number
Illumina pair-end	Illumina NovaSeq	19,573,148,100	223.20	SRR28000465
NanoPore	Oxford NanoPore	29,214,889,073	333.14	SRR28000457
Hi-C	DNBSEQ-T7	22,715,244,600	259.03	SRR28000066
TU_LabR1	Illumina NovaSeq	5,889,513,359	\	SRR28000928
TU_LabR2	Illumina NovaSeq	9,347,590,280	\	SRR28000929
TU_LabR3	Illumina NovaSeq	7,512,468,577	\	SRR28000930

### Genome survey

**Fig. 1:**
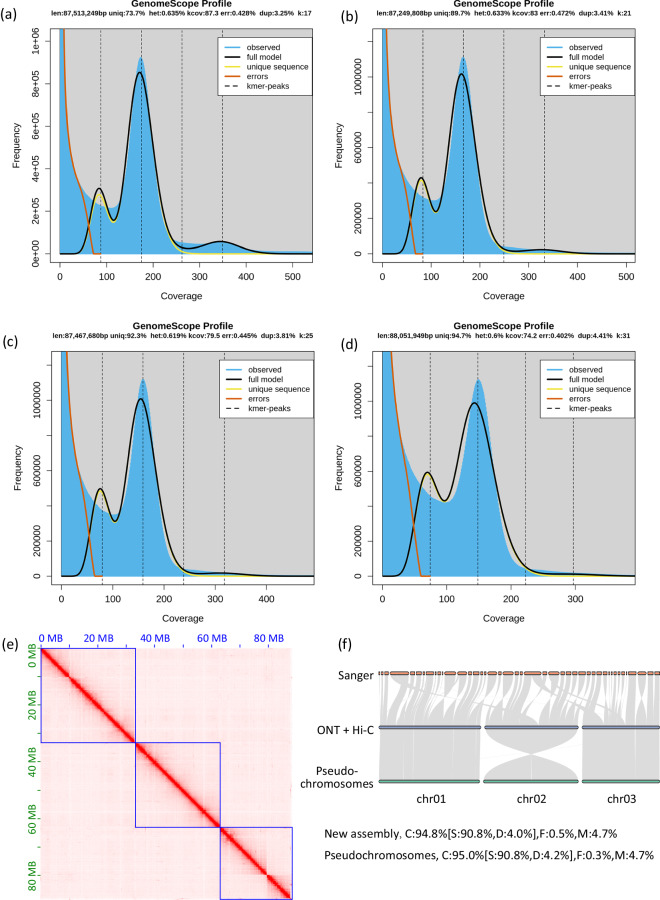
Genome survey and assembly of the two-spotted spider mite (TSSM) *Tetranychus urticae*. Genome size, heterozygosity and rate of duplication were estimated using Genomescope when k-mer = 17 (**a**), 21 (**b**), 25 (**c**), and 31 (**d**). (**e**) The genome-wide all-by-all Hi-C matrix of TSSM. Three linkage groups were identified based on Hi-C contacts, indicated by blue boxes. Sequences anchored on chromosomes are shown in the plot. (**f**) Synteny blocks between our new assembly and two previously published genome assemblies of TSSM.

Genome survey was performed using a k-mer based method. The k-mer coverage was counted from Illumina short reads using Jellyfish version 2.2.10^20^ with k-mers of 17, 21, 25, and 31. Genome size, heterozygosity, and duplication rate were estimated using GenomeScope version 2.0^21^. The estimated size of the TSSM genome rangs from 87.25 Mb to 88.05 Mb, with a heterozygosity rate of 0.60% to 0.64%, and a duplication rate of 3.25% to 4.41% (Fig. 1a–d).

### Genome assembly

Nanopore long-reads were corrected and assembled using Nextdenovo^22^ with default parameters. In order to remove possible secondary alleles, the assembled contigs were filtered using the pipeline *Purge Haplotigs*^23^, which produced 177 contigs with a total length of 103.35 Mb and a contig N50 of 3.46 Mb. Raw Illumina whole-genome short-reads were used to polish the long-read contig-level assembly using Pilon v1.22^24^. Hi-C Illumina short-reads were used to assemble contigs into a chromosome-level genome using Juicer v1.5^25^ and 3D-DNA^26^. The final assembly contains three chromosomes composed of 34 contigs with a total length of 87.7 Mb (Fig. 1e). This newly assembled genome has greater continuity, with 33 gaps, compared to a previously reported pseudochromosome-level genome, which consisted of 42 scaffolds with over 800 gaps^15,27^.

### Genome annotation

**Fig. 2:**
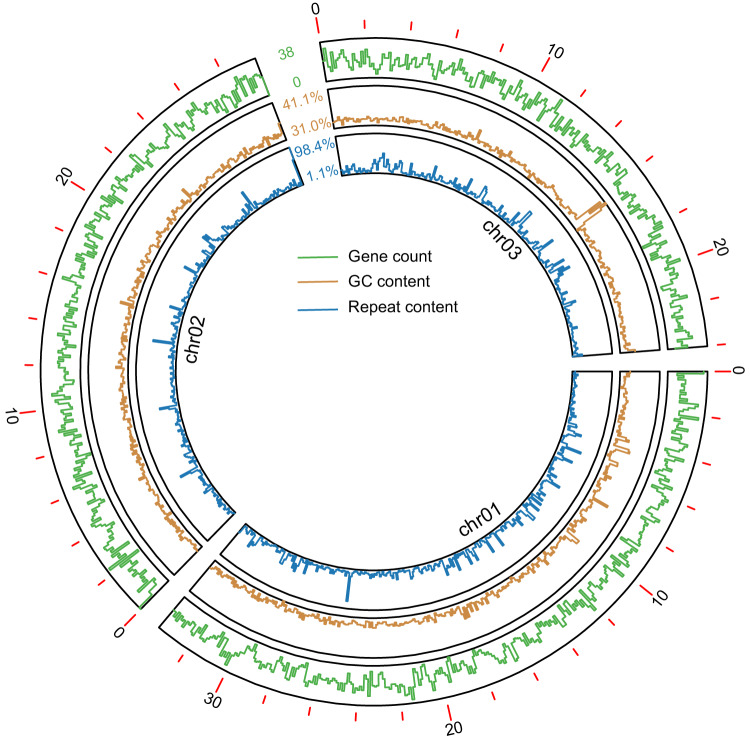
Circos plot of GC content, gene count, and repeat content of *Tetranychus urticae* genome.

The repeat annotation was performed with RepeatModeler v2.0.4^28^ and RepeatMasker v4.1.4^29^ using a species-specific repeat library, a RepBase database, and a repeat element library for Arthropoda from the Dfam database. The protein-coding genes were annotated using RNA-seq-based, *ab initio*, and homolog-based methods in the MAKER v3.01.04 pipeline^30^. For the RNA-seq-based method, the RNA-seq reads of three libraries were mapped to our TSSM assembly with Hisat v2.2.0^31^. The transcripts were then assembled using Stringtie v2.1.2. For *ab initio* annotation, SNAP v2013-02-16^32^ and Augustus v3.2.3^33^ parameters were estimated or trained before using them to predict genes in MAKER^30^. The SNAP parameters were estimated from high-quality transcripts obtained by improvement and filtering using PASA v2.4.1^34^. The gene model of Augustus was directly obtained from the above BUSCO analysis of the genome assembly. For the homolog-based method, we the used protein-coding genes of *Drosophila melanogaster* (dmel_r6.06) and the previously published genome of TSSM (Accession: GCF_000239435.1)^7^. Another homology-based method implemented in GeMoMa^35^ and transcript-based gene predictions utilized in the PASA pipeline v2.1.087^34^ were performed. Gene models from the three main sources were merged to produce consensus models by EvidenceModeler^36^. Finally, we identified 15,604 protein-coding genes, 11,232 of which were identical (>95%) to 10,725 protein sequences of the previous version^15^. Functions of the protein-coding genes were annotated using EggNOG-Mapper v2.1.7^37^ against the database EggNOG v5.0.2^38^, NR^39^, Swiss-Prot^40^, GO^41^, KEGG^42^, COG^43^ and PFAM^44^. In total, 11,435 genes could be functionally annotated. The gene count, Guanine-Cytosine(GC) content, and repeat sequence content were calculated in 100Kbp non-overlapping sliding windows using Bedtools v2.30^45^ and displayed in a Circos plot by TBtools v2.093^46^ (Fig. 2a).

## Data Records

Illumina short-reads, Nanopore, Hi-C raw reads for *T. urticae* genome sequencing and Illumina transcriptome data can be accessed in the NCBI Sequence Read Archive under project accession number PRJNA788385^47^, with accession numbers SRR28000465^48^, SRR28000457^48^, SRR28000066^48^ and SRR28000928- SRR28000930^48^, respectively. The finally assembled genome has been deposited in the NCBI with an accession number of JALDPR010000001-JALDPR010000051^49^. The genome assembly and annotation files are available in Figshare (10.6084/m9.figshare.25241794)^50^.

## Technical Validation

Completeness of the genome assembly was up to 94.8% (90.8% single-copied genes, 4.0% duplicated genes, 0.5% fragmented, and 4.7% missing genes) as assessed using BUSCO v3.0.2^51^ with the ‘arachnida_odb10’ database (n = 2934), similar to the previously assembled pseudochromosome-level genome (95.0% completeness with 90.8% single-copied genes, 4.2% duplicated genes, 0.3% fragmented, and 4.7% missing genes). The completeness for annotated gene set was 93.4% (87.1 single-copied genes, 6.3% duplicated genes, 0.7% fragmented, and 5.9% missing genes). Synteny between our assembly and a previously published assembly (the London strain, Assembly accession: GCF_000239435.1) as well as the chromosome-level reassembly^7,15^ was analyzed using MCSCAN^52^. This genome showed high synteny to a previously assembled scaffold-level and pseudochromosome-level genome (Fig. 1e). As noted by previous studies^15,27^, errors on scaffolds 1, 2, 4, and 8 of the Sanger assembly were resolved by our new assembly.

## Data Availability

No custom scripts or code were used in this study.
